# Neonatal evaluation by extended (12 area) vs. traditional (6 area) lung ultrasound scoring (NEXT-LUS): a prospective observational study

**DOI:** 10.3389/fped.2025.1638936

**Published:** 2025-08-13

**Authors:** Chinmay Chetan, Shoham Majumder, Aninda Debnath, Ravleen Kaur, Deepak Jaybhaye, Arshpuneet Kaur, Saikat Patra

**Affiliations:** ^1^ Himalayan Institute of Medical Sciences, Swami Rama Himalayan University, Dehradun, India; ^2^ All India Institute of Medical Sciences, Kalyani, India; ^3^ Maulana Azad Medical College, New Delhi, India

**Keywords:** preterm/full term infants, neonat*, lung, ultrasound, respiratory distress, invasive ventilation, respiratory outcomes, poCUS

## Abstract

**Background:**

Lung ultrasound (LUS) offers a safe, repeatable, radiation-free tool in management of respiratory distress in neonates. Despite wide use, limited data exists on optimal scoring approaches.

**Methodology:**

A prospective observational study was conducted over 6 months in a tertiary neonatal intensive care unit (NICU) enrolling neonates with respiratory distress within 2 h of admission after consent. LUS was performed using both 6-area and 12-area scanning approaches. Scores were assigned per Brat's criteria. Primary outcome was prediction of need for invasive ventilation within 72 h. Secondary outcomes included optimal cut-off scores, correlation with clinical outcomes and procedural safety.

**Results:**

Among 73 neonates enrolled, the 6-area LUS score (cut-off ≥5) predicted invasive mechanical ventilation within 72 h with 75% sensitivity and 67% specificity (AUC = 0.76). The 12-area score (cut-off ≥13) had similar accuracy (sensitivity 75%, specificity 73%; AUC = 0.77). Both 6-area and 12-area scores performed better in neonates <34 weeks (AUCs: 0.83 vs. 0.86). In neonates presenting after 24 h of life (*n* = 19), both scores maintained good accuracy (AUCs: 0.80 for 6-area, 0.83 for 12-area). Multivariate analysis identified partial pressure of carbon dioxide (pCO_2_) and duration of stay as independent predictors. The 12-area score required reattempts (in 9% cases) unlike the 6-area score.

**Conclusion:**

In neonates presenting with respiratory distress, 6-area and 12-area LUS scores done within 2 h of admission show good and comparable predictive value regarding need for invasive ventilation by 72 h.

## 1 Introduction

Respiratory distress is one of the most common indications of neonatal admission in neonatal intensive care unit (NICU) (1, 2). It requires rapid but thorough evaluation to guide prompt management and remains one of the most vexatious and exasperating challenges faced by neonatologists. Lung ultrasound (LUS) has emerged as a reliable, reproducible, non-invasive diagnostic tool for evaluating neonatal lung conditions (3–5). In contrast to the traditional chest x-ray, LUS is a radiation-free, point-of-care tool that can be repeated multiple times without any significant side-effects and provides “dynamic” assessment of neonatal lung (6). LUS is also quantifiable with standardized scores available to assess the severity of lung pathology (7). LUS scoring systems often utilize 12-area and 6-area approaches by assessing specific regions for sonological changes. While the 6-area approach offers a simplified method, assessing only three areas per hemithorax involving the anterior and lateral regions (7–9); the 12-area approach provides detailed evaluation by dividing each hemithorax into six areas taking the upper and lower half of anterior, lateral and posterior regions (10). The difference lies in manner of lung partitioning only. The degree of aeration is classified according to the same principles proposed by Brat et al. (7). An approach that includes scan of more areas including posterior regions ought to be theoretically more accurate as it allows for a more complete evaluation of lung regions. But both approaches have their advantages. Many of the common lung pathologies that manifest as respiratory distress often have findings that are localized to a specific area and may be missed in 6-area approach. Conversely, neonates are traditionally kept supine in most nurseries which do not allow ready access to the posterior areas included in the 12-area score. A comparison between the various methods of 6-area approach for prediction of the need for surfactant replacement showed that all three had very good predictive ability albeit at different cut-off scores and the differences among the scores were negligible (11). 6-area approach has been studied for prediction of short-term outcomes like extubation failure with contrasting results (12, 13). 12-area approach has also been used to predict severity of hyaline membrane disease and respiratory outcomes (14). 12-area approach has also been found to predict need for invasive respiratory support (15). Most of these studies are retrospective analyses. To the best of our knowledge, there are no studies directly comparing the 12-area vs. 6-area LUS approaches for predicting short-term outcomes in neonatal respiratory distress. Therefore, this study was designed to compare the diagnostic performance of the 12-area and 6-area LUS scoring systems in predicting these outcomes. By identifying the more effective scoring method, this research seeks to improve the accuracy and efficiency of respiratory distress management in neonates, ultimately enhancing quality of care in NICUs.

## 2 Materials and methods

### 2.1 Study design

It was a prospective observational study, conducted in the NICU of a tertiary care hospital of Northern India over a period of 6 months from October 2024 to March 2025.

### 2.2 Study population

All consecutively admitted neonates presenting in NICU for respiratory distress were included. Neonates with major congenital malformations, chromosomal anomalies, pulmonary hypoplasia, and air leaks (viz. pneumothorax, pneumomediastinum) that precluded comprehensive ultrasonological visualization of lung parenchyma, were excluded. Neonates in need of thoracic surgery during initial stay or those who were already intubated and on invasive ventilation prior to performance of LUS were also excluded.

### 2.3 Study procedure

Neonates presenting with a respiratory rate exceeding 60 breaths per min and/or exhibiting signs of chest retractions, nasal flaring, or grunting were enrolled in the study within two hours of admission, following informed consent from their guardians. Demographic data along with relevant maternal and neonatal clinical characteristics were collected and recorded on a structured proforma. Babies admitted to NICU were followed prospectively and details of management were recorded. LUS was performed within 2 h of admission with Sonosite Edge 2/P 20680, using linear probe with frequency of 6–13 MHz. Scans were performed in longitudinal (craniocaudal) planes. Depth and focus were adjusted according to patients' size and the sign of interest.

#### 2.3.1 For 12-area score

The chest surface was divided into three regions in each hemithorax with the anterior and posterior axillary lines as boundaries.

• Anterior region (from parasternal to anterior axillary line)
• Lateral region (from anterior to posterior axillary line)
• Posterior region (from posterior axillary to paravertebral line)

**Figure 1:**
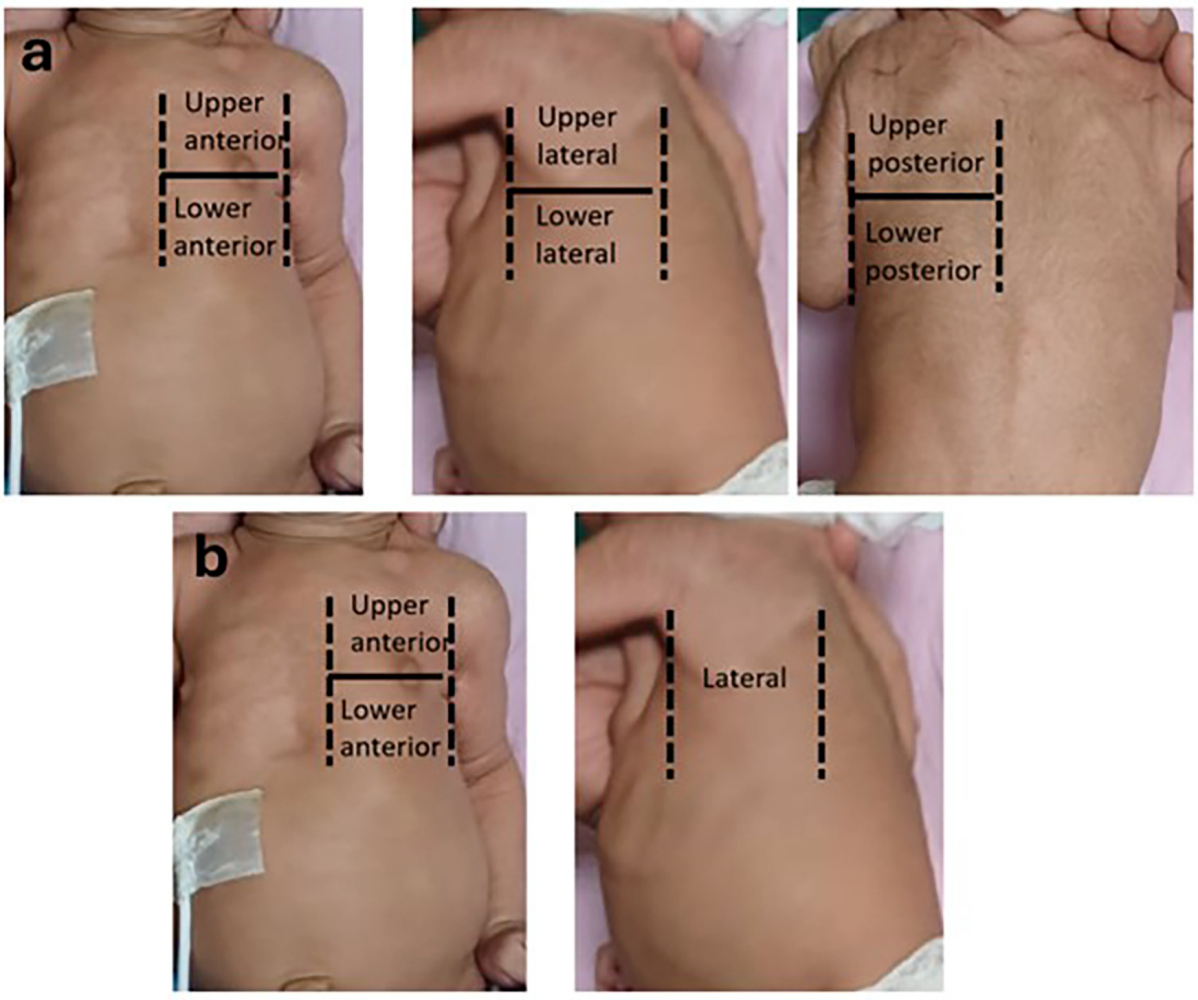
**(a)** 12 areas divided by anterior axillary line, posterior axillary line and line passing through the nipple. **(b)** 6-areas divided by anterior axillary line and line passing through the nipple.

Each region was divided into upper and lower areas by a line joining the nipples. Hence the total areas screened were right upper anterior (RUA), right lower anterior (RLA), right upper lateral (RUL), right lower lateral (RLL), right upper posterior (RUP), right lower posterior (RLP), left upper anterior (LUA), left lower anterior (LLA), left upper lateral (LUL), left lower lateral (LLL), left upper posterior (LUP), left lower posterior (LLP) (Figure 1a).

#### 2.3.2 For 6-area score

The chest surface was divided into three areas in each hemithorax by the anterior axillary line and a line passing through the nipple.

• Upper anterior area (from parasternal to anterior axillary line, above the nipple line)
• Lower anterior area (from parasternal to anterior axillary line, below the nipple line)
• Lateral region (from anterior to posterior axillary line)

Hence the total areas screened were right upper anterior (RUA), right lower anterior (RLA), right lateral (RL), left upper anterior (LUA), left lower anterior (LLA), left lateral (LL) (Figure 1b).

Each area was scored according to the degree of aerations as enumerated by the Brat et al. (7) (Table 1).

**Table 1:** Lung ultrasound score.

0	1	2	3
Only A lines, or <3 B lines	≥3 well-spaced B lines	Severe B pattern: confluent/compact B lines	Extensive consolidation >1 cm
Lung sliding present	A lines present/absent	Subpleural Consolidation <1 cm (Not an essential criteria)	Pleural effusion present/absent
	Lung sliding present	Minor consolidation/pleural effusion	
	No consolidation/pleural effusion		

The worst pattern for each area was documented for each neonate and the maximum score was summed up. The maximum possible score for both lungs is 36 for 12-area and 18 for 6-area approach. All lung ultrasounds were performed by experienced neonatologists, all of whom had at least three years of training and experience in point-of-care lung ultrasonography and at least one of whom was always present in the unit. The images were stored and independently scored by another consultant for cross-verification. In cases of scoring discrepancies, the images were reviewed by a senior radiologist, whose assessment was considered final. Neonates were managed in accordance with established unit protocols. Procedures including intubation, mechanical ventilation, and extubation were performed following standardized departmental guidelines (see Supplementary Material 1).

### 2.4 Outcome measures

The primary outcome was to compare the 12-area vs. 6-area LUS score to predict need of invasive ventilation in neonates with respiratory distress within 72 h of admission. Secondary outcomes included: identifying threshold score with highest area under curve (AUC) for 12-area and 6-area screening to predict need of invasive ventilation (within 72 h of admission), correlation of both scores with selected clinic-laboratory parameters and procedural safety during both approaches.

### 2.5 Sample size calculation

For an expected sensitivity of 79% and an expected specificity of 85% (16), taking the prevalence of invasive ventilation of 30% among neonates presenting in our NICU with respiratory distress based on data from last 12 months, the sample required to detect the predictive ability of LUS for invasive ventilation with the precision of 20%, confidence interval of 95%, were 54 and 18 respectively. Taking the larger sample size and with an expected drop rate of 20%, at least 65 neonates were to be recruited.

### 2.6 Statistical analysis

All statistical analyses were performed using the STATA, version 18. Descriptive statistics were used to summarize baseline characteristics, including frequencies and percentages for categorical variables, and means or medians with standard deviations or interquartile ranges for continuous variables, as appropriate. The primary diagnostic performance of the 6-area and 12-area LUS scores in predicting the need for invasive ventilation within 72 h was assessed using Receiver Operating Characteristic (ROC) curve analysis, and area under the curve (AUC) values were compared to determine discriminatory ability. Optimal cutoff scores were derived using Youden's Index. To evaluate associations between LUS scores and clinical outcome variables (e.g., maximum fraction of inspired oxygen (FiO_2_), partial pressure of carbon dioxide (pCO_2_), duration of stay), univariate Pearson correlation coefficients were calculated. Additionally, multivariate linear regression models were constructed separately for each LUS score to assess their independent predictive value after adjusting for clinical variables. A *p*-value <0.05 was considered statistically significant. All tests were two-tailed.

### 2.7 Study ethics

The study was approved by the institutional ethics committee of the Swami Rama Himalayan University, Dehradun, India. Neonates were enrolled after obtaining informed consent from their guardian(s).

## 3 Results

**Figure 2:**
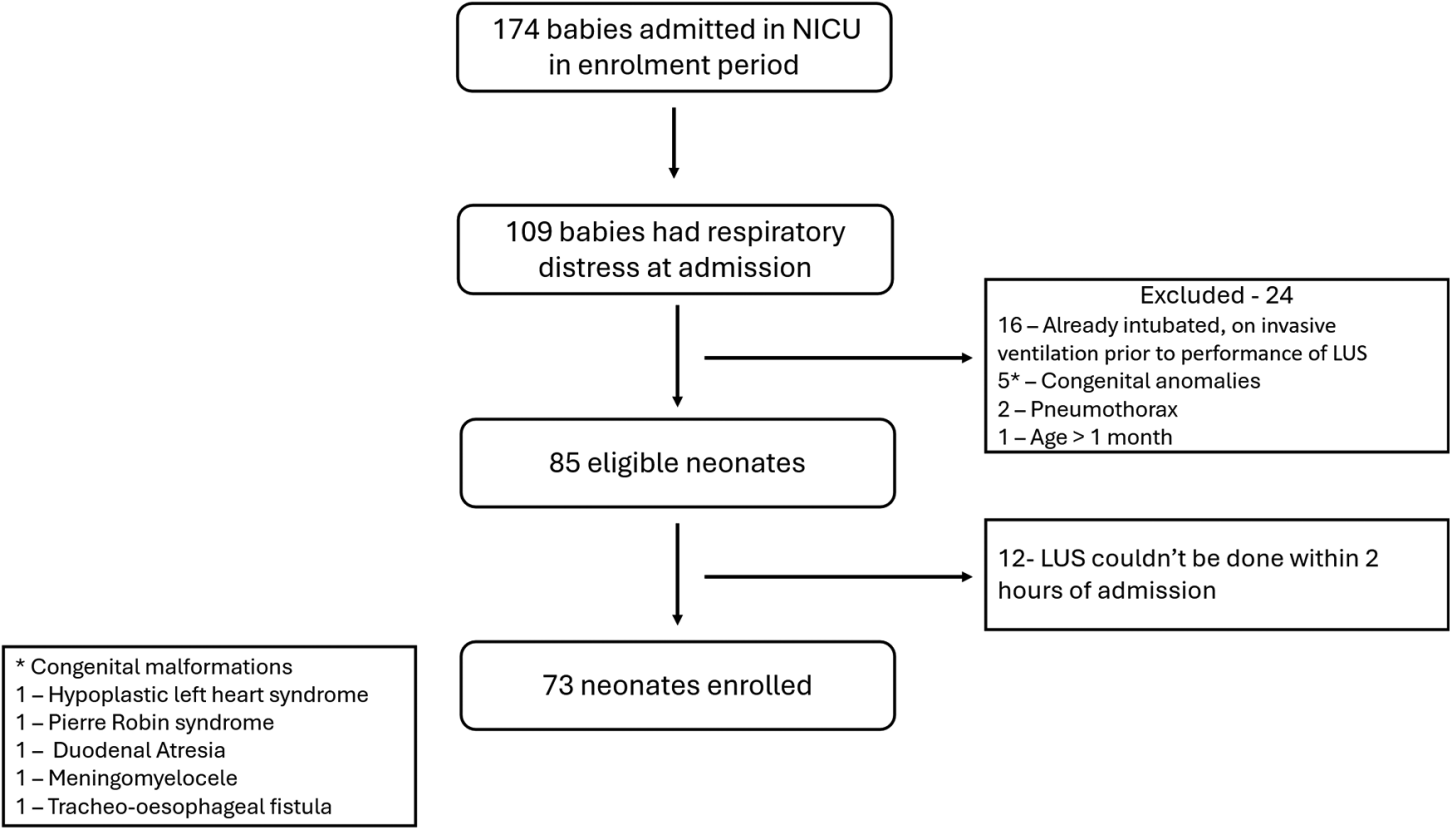
Study flow diagram.

174 neonates were admitted in the NICU during the study period, of whom 109 had respiratory distress at admission. 85 neonates were eligible. LUS couldn't be done within 2 h of admission in 12 neonates. A final of 73 neonates were enrolled in the study (Figure 2). At least one dose of antenatal corticosteroids were administered to 20 neonates, while 16 neonates received the complete course. Ten eligible neonates did not receive any antenatal steroid therapy. Among the 37 neonates born before 34 weeks of gestation, 13 received surfactant therapy, with 2 of them requiring a second dose. The baseline data in terms of relevant maternal and neonatal characteristics is given in Table 2.

**Table 2:** Baseline characteristics of the enrolled population.

Characteristic		*N* = 73
Maternal age (years)^a^		26.9 (4.2)
Mode of delivery^b^	Vaginal	26 (36)
	Operative	47 (64)
Gestational age^c^		33 (31–36)
Gestational age in weeks^b^	≥37	15 (20)
	34–36^6/7^	21 (29)
	32–33^6/7^	14 (19)
	< 32	23 (32)
Birth weight (grams)^c^		1,894 (1,400–2,384)
Birth weight in grams^b^	≥2,500	17 (23)
	1,500–2,499	34 (47)
	<1,500	22 (30)
Intrauterine status^b^	SGA	11 (16)
	AGA	59 (80)
	LGA	3 (4)
Downe's score at presentation^c^		4 (3–6)
Respiratory support at presentation^b^	HHHFNC	3 (4)
	CPAP	33 (45)
	NIV	14 (19)
	Invasive ventilation	23 (32)

^a^ Mean (Standard Deviation).

^b^ Number (percentage).

^c^ Median (interquartile range).

AGA, appropriate for gestation age; CPAP, continuous positive airway pressure; HHHFNC, heated humidified high flow nasal cannula; LGA, large for gestation age; NIV, non-invasive ventilation; SGA, small for gestation age.

**Figure 3:**
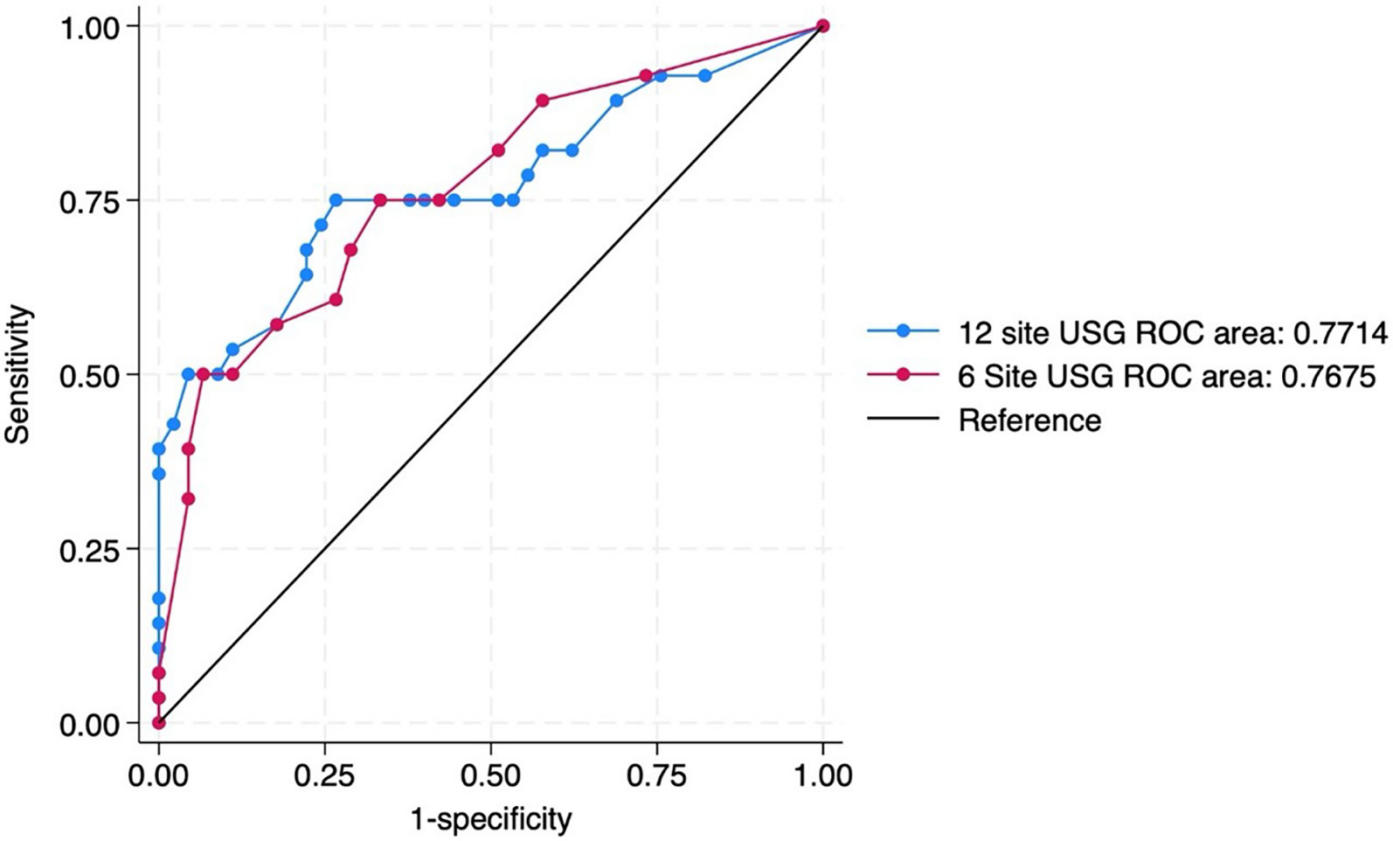
ROC curve predicting the need for invasive mechanical ventilation by 72 h of admission by 12 site USG and 6 site USG scores.

For primary objective the predictive ability of both the scores were compared. The need for invasive mechanical ventilation by 72 h of admission was predicted by 6-area LUS score with an optimal cut-off score of 5 (sensitivity 75% and specificity 67%) and by 12 area score with an optimal cut-off score of 13 (sensitivity 75% and specificity 73%). The area under ROC curves (AUC) were 0.76 and 0.77 respectively (Table 3) (Figure 3).

**Table 3:** AUC for predicting need for invasive mechanical ventilation by 72 h by 6-area score and 12-area score.

Group		AUC 6-area score	AUC 12-area score	*p*-value
Overall^a^		0.76	0.77	0.25
Gestational age at birth	<34 weeks	0.83	0.86	0.2
	≥34 weeks	0.67	0.64	0.4
Age at presentation	<24 h	0.77	0.76	0.59
	>24 h	0.80	0.83	0.24

^a^ Primary outcome.

**Figure 4:**
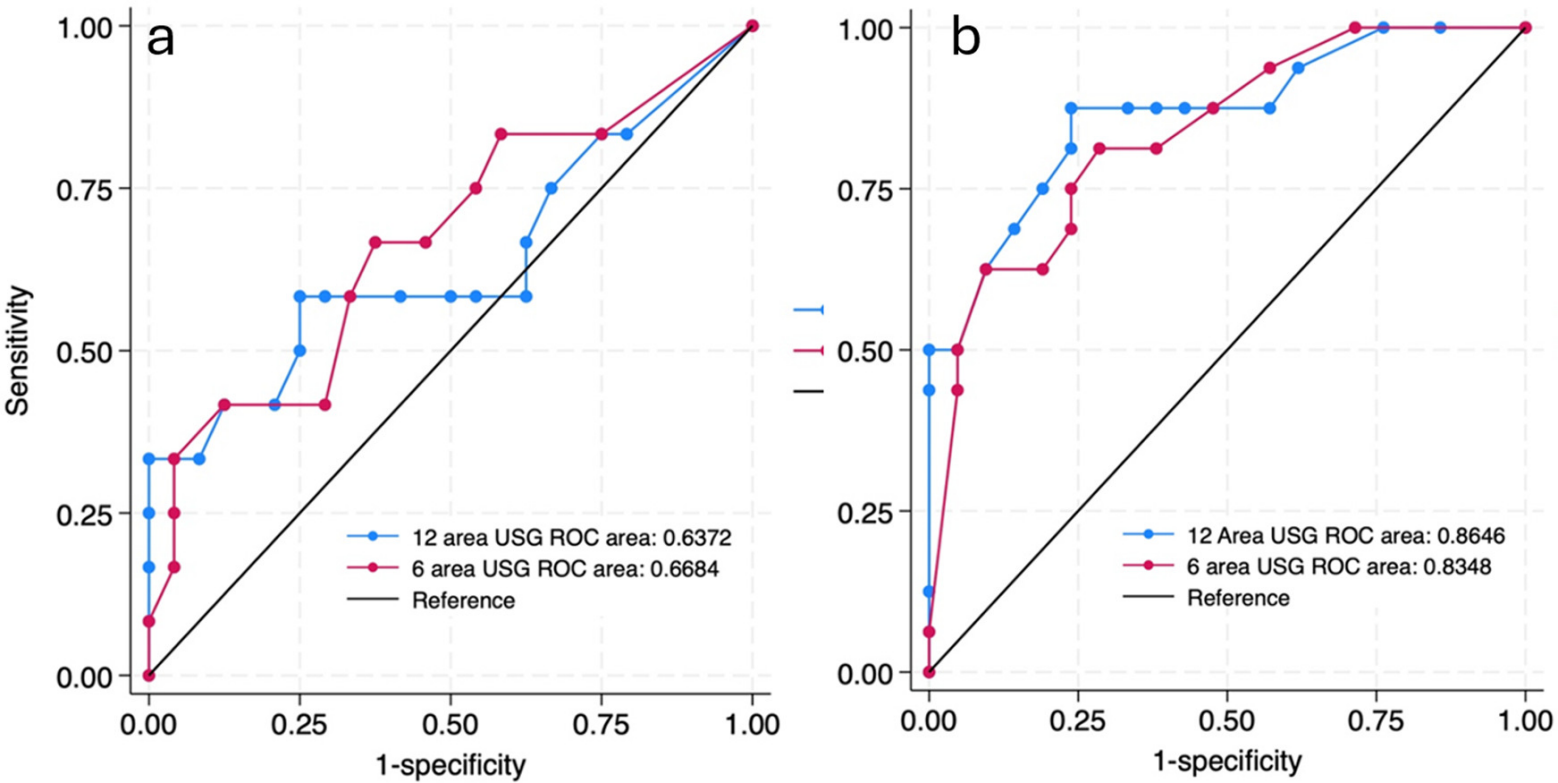
ROC curves predicting the need for invasive mechanical ventilation by 72 h of admission by 12 site USG and 6 site USG scores in ≥34 weeks **(a)** and <34 weeks **(b)** gestational age group.

Since respiratory pathologies often vary among neonates with gestational age <34 weeks compared to the rest, subgroup analysis was performed in this population. In neonates ≥34 weeks, the AUC for 6-area and 12-area approaches were 0.67 (0.46–0.87) and 0.64 (0.42–0.85) respectively. In neonates <34 weeks the predictive ability was higher with AUC for 6-area and 12-area approaches being 0.83 (0.70–0.96) and 0.86 (0.74–0.98) respectively (Figure 4). While both the scores had good predictive ability in neonates <34 weeks, the differences between them overall or in the gestational age subgroups were not statistically significant (Table 3).

Similarly postulating that lung pathologies presenting after 24 h of life would be more non-homogenous and likely to be differentiated by the two scores, such neonates (*n* = 19) were separately analyzed. There was no statistically significant difference between the AUC of the scores (0.80 and 0.83 respectively) (Table 3).

As shown in Table 4, univariate correlation analyses demonstrated that both the 6-area and 12-area LUS scores were significantly associated with key indicators of respiratory compromise. Specifically, both scores exhibited moderate-to-strong positive correlations with maximum FiO_2_ requirement (*r* = 0.430 and *r* = 0.497, respectively), maximum mean airway pressure (MAP) (*r* = 0.418 and *r* = 0.436), and maximum partial pressure of carbon dioxide (pCO_2_) (*r* = 0.552 and *r* = 0.550). Additionally, both scores correlated with the oxygen saturation index (OSI) and showed inverse relationships with worst arterial pH (*r* = –0.449 and *r* = –0.428), suggesting that higher LUS scores are associated with worsening acidosis. In contrast, neither score showed significant univariate correlations with cumulative hours of respiratory support or duration of NICU stay.

**Table 4:** Univariate correlation analysis between 6-area and 12-area LUS scores with key indicator of respiratory compromise and short-term outcomes.

Variable	6-area score	12-area score
Maximum FiO_2_ requirement	0.430 (0.000)	0.497 (0.000)
Maximum MAP	0.418 (0.000)	0.436 (0.000)
Maximum pCO_2_	0.552 (0.000)	0.550 (0.000)
Maximum OSI	0.455 (0.000)	0.492 (0.000)
Worse pH	−0.449 (0.000)	−0.428 (0.001)
Cumulative hours of respiratory support	0.080 (0.502)	0.151 (0.204)
Duration of NICU stay	0.007 (0.951)	0.048 (0.687)

FiO_2_, fraction of inspired oxygen; MAP, mean airway pressure; OSI, oxygen saturation index; pCO_2_, partial pressure of carbon dioxide.

Multivariate regression analysis of the 6-area LUS score (Table 5) revealed several statistically significant associations even after adjusting for other variables. The overall model was significant (F = 5.091, *p* < 0.001), with an *R*^2^ of 0.464, indicating that approximately 46% of the variance in clinical outcomes could be explained by the 6-area score. The strongest independent predictor was maximum pCO_2_ (*β* = 0.148, *p* = 0.001), reinforcing the clinical validity of the score in identifying neonates with impaired ventilation. Additionally, the 6-area score was significantly associated with duration of NICU stay (*β* = 0.346, *p* = 0.011), implying that higher LUS scores are linked to longer hospitalization. A marginal association with worse pH (*β* = 9.56, *p* = 0.096) was also observed, further supporting the score's relationship with respiratory acidosis. Notably, FiO_2_ requirement, MAP, OSI, and respiratory support hours were not statistically significant in the multivariate context.

**Table 5:** Multivariate regression analysis of 6-area LUS score with key indicator of respiratory compromise and short-term outcomes.

6 area score	Correlation coefficient	Standard error	*P* value	95% CI
Maximum FiO_2_ requirement	−0.02	0.05	0.71	−0.13 to 0.09
Maximum MAP	0.34	0.49	0.5	−0.66 to 1.33
Maximum pCO_2_	0.15	0.04	<0.01*	0.06–0.23
Maximum OSI	0.07	−0.49	0.89	−0.91 to 1.04
Worse pH	9.56	5.63	0.09	−1.76 to 20.89
Cumulative hours of respiratory support	−0.79	0.94	0.40	−2.67 to 1.09
Duration of NICU stay	0.35	0.13	0.01*	0.08–0.60

FiO_2_, fraction of inspired oxygen; MAP, mean airway pressure; OSI, oxygen saturation index; pCO_2_, partial pressure of carbon dioxide; CI, confidence interval.

The corresponding multivariate regression for the 12-area score (Table 6) yielded similar findings but demonstrated slightly weaker model performance overall. The model was significant (*F* = 4.681, *p* < 0.001), with an *R*^2^ of 0.443. Like the 6-area score, the 12-area score was independently associated with maximum pCO_2_ (*β* = 0.296, *p* = 0.002) and duration of NICU stay (*β* = 0.685, *p* = 0.017). However, association with FiO_2_, MAP, OSI, and pH was not statistically significant.

**Table 6:** Multivariate regression analysis of 12-area LUS score with key indicator of respiratory compromise and short-term outcomes.

12 area score	Correlation coefficient	Standard error	*P* value	95% CI
Maximum FiO_2_ requirement	0.02	0.12	0.88	−0.21 to 0.25
Maximum MAP	0.41	1.05	0.69	−1.70 to 2.52
Maximum pCO_2_	0.29	0.09	<0.01*	0.12–0.47
Maximum OSI	0.05	1.03	0.96	−2.04 to 2.13
Worse pH	17.08	11.97	0.16	−6.99 to 41.15
Cumulative hours of respiratory support	−1.19	1.99	0.55	−5.20 to 2.81
Duration of NICU stay	0.68	0.27	0.01*	0.13–1.24

FiO_2_, fraction of inspired oxygen; MAP, mean airway pressure; OSI, oxygen saturation index; pCO_2_, partial pressure of carbon dioxide; CI, confidence interval.

While doing 12-area scoring, attempts had to be interrupted or aborted midway and reattempt for scan made in 7 babies (9%) while there was no need for any reattempt with 6-area scoring. All the episodes of interruptions were due to desaturation. The desaturations resolved spontaneously and required no additional interventions. There were no episodes of bradycardia or other clinical instabilities while performing any of the scans.

## 4 Discussion

Our study revealed no significant differences between 12-area and 6-area approach in terms of predictive ability for short term respiratory outcomes. Both the scores were able to predict the need for mechanical ventilation by 72 h of admission with an AUC of more than 0.75. The ROC analysis underscores key thresholds in both LUS scoring systems that can meaningfully inform NICU decision-making. For the 6-area score, sensitivity remains high up to a score of 4 (75%–93%), but specificity gradually improves, suggesting this range may be most useful for early screening. A score of 5 offers an optimal balance with 75% sensitivity and 66% specificity, making it a well-suited practical threshold for early identification of neonates at risk of requiring invasive ventilation. Beyond a score of 7, specificity increases sharply (73% at score 7, 82% at score 8, and 93% at score 10), but at the cost of reduced sensitivity (<60%). For the 12-area score, although a cut-off of ≥12 offers similar balance, a threshold of ≥13 emerges as more clinically advantageous—retaining sensitivity at 75% while improving specificity to 73%. This enhances the ability to detect true positives without overtriaging, thus minimizing unnecessary interventions. Higher cut-offs (≥14) improve specificity further but at the cost of reduced sensitivity, potentially delaying treatment in at-risk neonates. At optimal cut-offs (score ≥5 for the 6-area and score ≥13 for the 12-area), sensitivity was identical (75%) for both, while specificity was slightly higher for the 12-area score (73%) compared to 67% for the 6-area. The overall area under the curve (AUC) was 0.76 for the 6-area and 0.77 for the 12-area approach, indicating comparable discriminative ability. The finding that both 6-area and 12-area LUS scores have similar AUCs for predicting imminent invasive ventilation suggests that a simplified examination may suffice for clinical decision-making. The similar sensitivity (75%) and close specificity (67% vs. 73%) between scoring methods reinforce that the shorter exam did not substantially compromise true-positive or true-negative rates. Clinicians aiming to quickly stratify risk at the bedside could rely on a 6-area protocol (requiring fewer probe positions and less scanning time) without a meaningful loss in predictive accuracy.

Neonates presenting below 34 weeks of gestational age with respiratory distress usually have higher incidence of hyaline membrane disease, a homogenous lung pathology. Late preterm and term babies usually have lung pathologies with more localized and non-homogenous afflictions. Hence subgroup analysis was done in these two gestational age groups. Subgroup analyses by gestational age (<34 weeks vs. ≥34 weeks) revealed that predictive performance was robust in the <34 weeks group (AUC: 0.83 for 6-area, 0.86 for 12-area). In contrast in neonates ≥34 weeks, accuracy dropped (AUC: 0.67 vs. 0.64, respectively) likely reflecting the heterogeneous aetiologies (transient tachypnea, meconium aspiration, early-onset pneumonia) and more focal lung involvement in this subgroup. Similarly, lung diseases presenting within the first day of life have more homogenous affectation of all the lung regions while pathologies localize to posterior regions after 24 h. Most of the neonates in our study presented within the first 24 h of life. In neonates presenting after 24 h of life (*n* = 19), both scores maintained good accuracy (AUC 0.80 for 6-area, 0.83 for 12-area), and differences did not reach statistical significance.

In both regression models, maximum pCO_2_ emerged as the strongest independent predictor of LUS scores. This aligns with the pathophysiological notion that poor alveolar ventilation and atelectasis—manifested sonographically as coalescent B-lines and consolidations—drive hypercarbia. The persistent association with pCO_2_ after adjusting for FiO_2_ and MAP suggests that LUS reflects the extent of alveolar filling or collapse rather than simply oxygenation impairment. Higher LUS scores independently predicted prolonged NICU stay. LUS assessment within two hours of admission can serve as a marker for overall severity trajectory—enabling prognostication, resource and personnel allocation, and help in early family counselling. Seven neonates (9%) undergoing the 12-area exam experienced desaturation episodes that necessitated interruption and re-attempting the scan, whereas no adverse events occurred during 6-area imaging. The neonates with desaturation recovered spontaneously and required no additional interventions. The lateral positioning of the neonate and additional probe placements required by the 12-zone protocol might have increased the risk of transient oxygen desaturation. Given the similar predictive performance, the 6-area protocol can minimize procedural risk without sacrificing diagnostic accuracy.

The lung was traditionally considered a poor candidate for ultrasonography due to high acoustic impedance by the air inside. The pathbreaking work by Lichtenstein et al. in 1980s demonstrated that previously neglected artifacts in LUS could provide critical real-time information about myriads of lung pathologies (17). A litany of LUS semiotics has now been standardized and validated across age groups (18). These are more readily recognizable in neonates because of lack of obesity or heavy musculature. To objectify LUS evaluation, Brat et al. modified an index proposed in adults for grading of degree of aeration (19). A-lines in LUS represent pleural reverberation through air filled lungs while B-lines are due to fluid content in the interstitial space. As the aeration worsens, A-lines reduce while B-lines increase in number and confluence, and the score increases (7). While this scoring pattern has been followed near universally in neonatal studies, the areas of lung scanned, and the pattern of lung partitioning have varied widely. Most of the initial studies used 6-area approach. Brat et al. used antero-superior, antero-inferior and lateral areas in each hemithorax (7). This is commonly practiced and has been referred as the “classical” approach. Raimondi et al. on the other hand partitioned lung into six areas based on midclavicular, anterior axillary and posterior axillary lines in each hemithorax, eschewing any superior/inferior division (8). Rodriguez-Fanjul et al. also utilized 6 areas and avoided superior/inferior divisions but partitioned each hemithorax into anterior, lateral and posterior regions (9). Extended LUS (eLUS) protocols have scanned 10 regions of lungs by adding bilateral postero-superior and postero-inferior regions to the classical approach. eLUS has been validated for prediction of surfactant requirement and predicting the respiratory course in preterm infants in the multicenter SLURP study (20). The 12-area approach additionally subdivides the lateral area also into upper and lower divisions creating the most extensive scanning protocol. The main difference between the classical and the 12-area approach is however the addition of the posterior regions. In sick neonates, the posterior areas are likely to be the dependent parts most of the time. Consequently, many pulmonary pathologies preferentially involve the posterior areas of the lung due to the gravitational effect. Non-homogenous lung diseases like meconium aspiration syndrome may also significantly involve posterior regions (21). Hence a 12-area approach should provide a more comprehensive picture of lung pathologies. Our study, designed to compare the extensive scanning approach with the classical scanning approach, however failed to show any significant differences between both approaches. One of the reasons could be that most of our enrolled babies were scanned within the first few hours of life when the lung pathologies demonstrate mostly homogenous picture. Over time the aeration improves in the anterior region and poorer scores are localized to posterior areas (22). Though we performed a subgroup analysis in neonates presenting after 24 h which showed no difference between the scoring approaches, the number (*n* = 19) was probably too small to capture significant difference. Studies adequately recruiting neonates presenting after 24 h for respiratory distress might be better able to elucidate this crucial distinction.

This is the first study, to our knowledge, which has compared the classical and widely practiced 6-area approach with the extensive 12-area approach for predicting short term respiratory outcomes. Previously, studies have attempted to individually assess the various scoring approaches. Aiswarya et al. in their study, using 6-area LUS approach predicted the need for intubation and invasive ventilation in neonates admitted in the NICU for respiratory distress. With the cut off of 7 or more, the LUS predicted the need for intubation with sensitivity of 79.17% and specificity of 84.42% with area under curve (AUC) of 0.838. It is almost similar to present study where the optimal cut-off score was 5 for 6-area approach with comparable sensitivity but less specificity. The differences could be due to difference in demographics with more preterm neonates recruited in that study (16). Zhang et al., using the 12-area scoring method, reported that a LUS cut-off score of 8 predicted the need for invasive respiratory support in neonates under 32 weeks' gestation with an AUC of 0.863. At this threshold, the sensitivity and specificity were 74% and 68.3%, respectively. For neonates of 32–36 weeks gestation, AUC was 0.863, with a cut-off score of 7, and sensitivity and specificity were 75.3% and 83.6%, respectively (15). Abdul Razak et al. reported pooled sensitivity 88% and specificity 82% (cut off LUS >5–6) for surfactant or mechanical ventilation in infants on CPAP (23). De Luca et al. used a 6-area LUS in late preterm and term infants and found that a score >8 predicted surfactant need with an AUC of 0.87 (95% CI 0.81–0.92), sensitivity 81%, and specificity 81%. Notably, they observed no significant difference in AUC between late preterm and term subgroups, suggesting consistent LUS utility across gestational ages (24). In contrast, extended-zone protocols (adding posterior fields or using more lung subdivisions) have sometimes shown marginal gains. Pang et al. used the 12-zone score and found that for predicting invasive ventilation the 12-zone LUS had AUC 0.912, sensitivity 81.3%, and specificity 88.8% (cutoff 25.5). This differs from our findings. The difference is probably due to more preterm babies being enrolled in that study (mean gestational age of 29 weeks and birth weight of 950 grams compared to 33 weeks and 1,894 grams in current study) (25). Szymański et al, in their study on preterm infants (< 32 weeks gestation) with RDS used a modified LUS score (4 area approach) which included posterior instead of lateral lung fields, and a 5-grade rating scale instead of a 4-grade rating scale. It showed that LUS score done within 24 h of birth had high reliability in predicting need for invasive ventilation on day of life 3 (AUC = 0.845) with the cut off score varying with the birth weight (26). Overall, these extended LUS scoring studies generally report AUCs in the high 0.8–0.9 range for predicting surfactant or invasive ventilation. However, head-to-head comparisons are sparse. Few studies have reported the procedural issues while performing extended scans. While all ultrasound evaluations are bound to be operator dependent, most studies have reported excellent concordance (27) and short learning curves (28).

The strengths of this study include its prospective nature. The population included cross-section of inborn and referred neonates presenting with varying severity of respiratory distress. The study has used commonly available probe, and imaging has been done by experienced personnel trained in LUS strictly within the set time frame. Inter-operator subjectivity inherent to any ultrasonography was sought to be mitigated through extensive training and high interobserver agreement coefficient was observed. Confirmation was done by sonologist in case of discrepancies.

This study has some limitations. It was conducted at a single centre, which may limit the generalizability of the findings. The study population primarily comprised moderate and late preterm neonates, with limited representation of extremely preterm infants. Additionally, all lung ultrasound examinations were performed by operators with over three years of experience. These factors should be considered when interpreting the applicability of the results to other clinical contexts. The clinicians treating the neonates were not blinded to the LUS scores. The LUS was performed once within 2 h of admission. Because LUS is quick and non-ionizing, the usefulness of repeated sequential scans (for e.g., at 2, 6 and 12 h) is an avenue that can be studied. Further studies may also try to compare the two approaches in neonates presenting after 24 h of life. 12 neonates could not undergo LUS within the defined time frame, potentially introducing bias. The study was geared towards short-term respiratory outcomes and long-term respiratory outcomes (e.g., chronic lung disease at 36 weeks postmenstrual age) were not assessed, limiting our ability to link early LUS scores with downstream morbidity. Since the traditional imaging techniques like chest x-ray and computerized tomography scans are scarcely easy to perform at bedside, LUS will continue to be studied and used for wider indications in NICU. Effective training in LUS for neonatologists necessitates the development of standardized protocols. Comparative studies of different scanning approaches will be essential to identify the most effective approaches and to inform the refinement of these protocols.

## 5 Conclusion

Both 6-area and 12-area approaches for lung ultrasound scoring had good ability for predicting short term outcomes in neonates with respiratory distress and provide reliable early prognostic information regarding the need for invasive ventilation, pCO_2_ levels, and length of NICU stay. The lack of significant difference persists albeit to varying degrees in subgroup analysis, whether neonates are born before or after 34 weeks gestational age and whether presenting within or after 24 h of life. Given its equivalent predictive accuracy and lower procedural burden, a 6-area LUS protocol emerges as a safe bet despite the presence of more extensive 12-area approach, particularly in those at higher risk of desaturation. Integrating LUS assessments into standard NICU admission workflows can enhance real-time risk stratification and guide prognostication.

## Data Availability

The raw data supporting the conclusions of this article will be made available by the authors, without undue reservation.
