# Supplementary material for: Neonatal evaluation by extended (12 area) vs. traditional (6 area) lung ultrasound scoring (NEXT-LUS): a prospective observational study
Source: Front Pediatr. 2025 Aug 13;13:1638936. doi: 10.3389/fped.2025.1638936 (PMC12380791; doi:10.3389/fped.2025.1638936)
Supplement: Supplementary file 1 [file Table1.docx]

**Unit protocol for management of babies with respiratory distress**

The neonates were managed according to the standard neonatal care guidelines and unit protocols.

1. Intubation and invasive ventilation were done for the following indications.

- Baby on non-invasive respiratory support. Still having
  - Frequent apneas (> 3/ hour or any episode requiring bag and mask ventilation).
  - Requirement of FiO_2_ > 0.6–0.7, PaO2<40 mmHg.
  - ABG showing PaCO_2_ > 60–65 mm Hg with persistent acidosis (pH<7.20).
- Clinical judgement of treating neonatologist

1. The babies were ventilated and nursed using standard NICU protocols. Extubation readiness was assessed in daily morning and evening rounds. Planned extubation was done for the following indications:

- FiO_2_ requirement < 40 %
- MAP < 8 – 10 cm H_2_O
- Mandatory breaths of the ventilator < 30/minute
- Presence of adequate spontaneous breathing
- Hemodynamically stable (not more than 1 inotrope)
- Last hemoglobin > 10 g/dl
- ABG while not mandatory, if done (2 hours prior to extubation)
  - No metabolic acidosis (pH>7.2)
  - PaCO_2_ - < 55mmHg, PaO_2_ > 40 mmHg
- Clinical Judgement of treating physician

1. Post-natal steroids were considered if the baby with gestational age ≤ 32 weeks required respiratory support after 28 days of life due to bronchopulmonary dysplasia.
2. Discharge criteria

- Gaining weight for at least 3 consecutive days on breastfeeding/ad libitum katori spoon feeds.
- Maintaining temperature with appropriate clothing.
- Maintaining target saturation with no distress on room air.
- Hemodynamically stable.
- Off injectable medications.
- Mother/guardians confident in taking care and trained for giving medications.
